# Overexpression of Tobacco *GCN2* Stimulates Multiple Physiological Changes Associated With Stress Tolerance

**DOI:** 10.3389/fpls.2018.00725

**Published:** 2018-06-01

**Authors:** Ning Li, Song-jie Zhang, Qi Zhao, Yue Long, Hao Guo, Hong-fang Jia, Yong-xia Yang, Hong-ying Zhang, Xie-feng Ye, Song-tao Zhang

**Affiliations:** Tobacco Cultivation Key Laboratory of China Tobacco, College of Tobacco Science, Henan Agricultural University, Zhengzhou, China

**Keywords:** GCN2, eIF2α kinase, stress resistance, ROS, *Nicotiana tabacum*

## Abstract

General control non-derepressible-2 (GCN2) is a ubiquitous protein kinase that phosphorylates the α subunit of the eukaryotic initiation factor, eIF2, preventing the initiation of a new cycle of protein synthesis, subsequently reducing the global protein biosynthesis. GCN2 can also regulate the response of plants to biotic and abiotic stresses. In this study, two GCN2 homologs, *NtGCN2-1* and *NtGCN2-2*, were cloned from *Nicotiana tabacum*, and were predicted to have been derived from their progenitors in *N. tomentosiformis* and *N. sylvestris*, respectively. The phosphorylation of NteIF2α could be activated by promoting the expression of *NtGCN2* with plant hormones, including salicylic acid (SA), azelaic acid (AZA), methyl jasmonate (MeJA), and by imposition of different stresses (*Bemisia tabaci* infection, drought, and cold), indicating that *NtGCN2* is involved in the response of plants to multiple biotic and abiotic stresses. We also observed that the overexpression of *NtGCN2-1* significantly influenced different physiological processes. It promoted seed germination and root elongation. The content of total soluble sugars and reducing sugars were decreased, whereas those of chlorophyll a and b were increased in the GCN2 overexpressing plants. In addition, the overexpressing plants had lower content of reactive oxygen species and exhibited higher antioxidant activities. These physiological alterations could be attributed to the changes in the endogenous phytohormones, decrease in the SA and abscisic acid content, and accumulation of MeJA and AZA. It indicated that the overexpression of *NtGCN2* in tobacco, stimulated the plant defense responses via phosphorylation of NteIF2α and regulation of plant hormones, and changes in the antioxidant ability and plant nutrient status.

## Introduction

General control non-derepressible-2 (GCN2) is a protein kinase that phosphorylates the α subunit of eukaryotic translation initiation factor 2 (eIF2α) during the protein synthesis process, resulting in the inhibition of conversion of eIF2γ-GDP to eIF2γ-GTP, and thus, preventing the initiation of a new cycle of translation, which reduces the global protein synthesis (Wek et al., 2006).

GCN2 is ubiquitous in eukaryotes. It was first reported and studied in yeast, in which GCN2 specifically phosphorylated the α subunit of eIF2 and could be activated under conditions of nutrient deprivation (amino acid and purine starvation) and salt stress (Dever et al., 1992; Goossens et al., 2001). The serine residue at position 51 (Ser-51) of eIF2α was shown to be critical for GCN2-dependent phosphorylation. When Ser-51 was substituted with alanine, the increased phosphorylation in response to amino acid deficiency was completely eliminated and the expression of GCN4, which is a master regulator of gene expression in yeast, was decreased to the same extent as was observed in the case of GCN2 deletion (Hinnebusch, 1997). cpc-3, a *Neurospora crassa* homolog of yeast GCN2, encodes a polypeptide with juxtaposed eIF2α kinase and histidyl-tRNA synthetase-related domains. The cpc-3-disrupted strain failed to induce an increase in transcription and derepression of amino acid biosynthetic enzymes in response to amino acid starvation (Sattlegger et al., 1998).

A homolog of yeast GCN2 was also isolated from *Drosophila melanogaster*. It was shown to contain a protein kinase catalytic domain and a histidyl-tRNA synthetase domain, and showed ability to complement the yeast *gcn2* mutant (Olsen et al., 1998). Four types of eIF2α kinases (PKR, PERK, HRI, and GCN2), which respond to different kinds of stimuli, are known to exist in mammals. Among these, GCN2 is important for translational control by eIF2α phosphorylation that leads to a global inhibition of protein synthesis. The phosphorylation of eIF2α acts as a molecular switch that shifts cells from proapoptotic to cytoprotective state in response to prolonged glucose deficiency. The catalysis of eIF2α phosphorylation by GCN2 plays a proapoptotic role in response to glucose deficiency (Muaddi et al., 2010).

Although GCN2 homologs are widely present in plants, as revealed by the results of protein BLAST done using yeast GCN2 sequence as a query, most of the studies have been focused on *Arabidopsis thaliana*, in which only one GCN2 has been identified, which shares approximately 50% sequence similarity with its homolog in yeast (Zhang et al., 2003, 2008; Lageix et al., 2008; Li et al., 2013). Sequence analysis showed that AtGCN2 contains five conserved domains that are crucial for its functions. Transcriptional profiling revealed that *GCN2* is expressed in roots, leaves, stems, and flowers in higher plants (Zhang et al., 2003, 2014). Heterologous expression in yeast demonstrated that AtGCN2 could complement the yeast *gcn2* mutant strain under amino acid deprivation (Zhang et al., 2003).

GCN2 acts as an important regulator in stress-response pathway in plants. It phosphorylates eIF2α, thereby, preventing the reinitiation of protein synthesis; thus, the presence of GCN2 may help the cell in coping with different stresses (Baena-Gonzalez, 2010; Li et al., 2013). A variety of biotic and abiotic stresses, including UV irradiation, methyl methanesulfonate (MMS), and chlorsulfuron treatment, cold shock, and wounding, can induce the GCN2-mediated phosphorylation of eIF2α (Lageix et al., 2008; Zhang et al., 2008). The activation of AtGCN2 by wounding, or by exposure to methyl jasmonate (MeJA) and salicylic acid (SA), suggested that eIF2α kinase could play a role in plant defense against insect herbivores (Lageix et al., 2008). GCN2 also functions in seed germination, regulation of leaf shape, and maintenance of the chlorophyll content. Loss of GCN2 functions in the *atgcn2* mutant was reported to result in lower germination rate with or without prior stratification, different leaf morphology, and higher chlorophyll accumulation (Liu et al., 2015). In addition, GCN2 was identified as a cellular component that supports the action of glyphosate *in vivo*. A deficiency of AtGCN2 minimized the effects of herbicide by compromising the molecular program that plants deploy after glyphosate treatment (Faus et al., 2015). Generally, GCN2 functions conservatively as an amino acid sensor in eukaryotes, from human to plants, and could be activated by uncharged tRNAs under conditions of amino acid deficiency (Dong et al., 2000; Towle, 2007; Li et al., 2013). Recent studies have shown that GCN2 does not sense the cysteine levels, whereas it was specifically activated by the C/N supply for cysteine production through an unknown mechanism (Dong et al., 2017). In addition, the GCN2 pathway, in concert with the TOR-autophagy pathway, might play an important role in the maintenance of amino acid homeostasis, and can independently contribute to branched-chain amino acid (BCAA) deficiency (Zhao et al., 2018). Although studies on GCN2 have made great progress in *Arabidopsis*, its function and response to various stresses have not been well studied in tobacco.

In this study, we cloned two GCN2 homologs from *N. tabacum*, which is a good model for understanding the mechanism of abiotic stress tolerance (Schaeffer et al., 2012). To characterize their functions in plants, we adopted a different strategy from that used in previous studies where GCN2 was down-regulated. The changes in the expression of *NtGCN2* under various biotic and abiotic stresses and the effects of its overexpression in tobacco were investigated. These results will help us in better understanding of the contribution of GCN2 in the response of plants to different stresses.

## Materials and Methods

### Plant Materials and Growth Conditions

*Nicotiana tabacum* (K326) seeds were germinated on soil or solidified Murashige and Skoog (MS) medium containing 1% (w/v) sucrose and were cultivated in a growth chamber at 28°C under a 16-h light/8-h dark cycle. To select the transformed seedlings, T1 transgenic plants were grown on MS medium supplemented with 100 μg/mL kanamycin.

### Treatment of Plants

Salicylic acid, azelaic acid (AZA), and MeJA were dissolved in aqueous solutions containing 5 mM 2-[*N*-Morpholino] ethane-sulfonic acid (MES) (pH 5.6) and 0.01% Tween-20. The SA, AZA, and MeJA solutions were used at working concentrations of 1 mM. Tobacco leaves were sprayed with the solutions until liquid started dripping off from their surface. For drought treatment, watering of 6-week-old tobacco plants was withheld for 0 (RWC = 60%), 3 (RWC = 30%), 6 (RWC = 18%), and 9 (RWC = 10%) days. For cold treatment, 10-days-old seedlings grown on MS plates were kept at 4°C in a growth chamber. After treatment, leaves were collected at the indicated times for analysis. For *Bemisia tabaci* infection, experiment was performed as described (Gdjr et al., 1983). Tobacco seedlings with 5–7 leaves were exposed to 100 whitefly adults (female:male = 1:1) in a cage (1 m × 0.8 m × 0.8 m) kept in a growth chamber. The leaves of the tobacco were collected at 24, 48, and 72 h after releasing the whitefly, and were immediately frozen in liquid nitrogen.

### Seed Germination, Seedling Growth, and Treatment

About 100 tobacco seeds of similar size were placed on a water-soaked filter paper in Petri dishes and cultured in a growth chamber at 28°C under a 16-h light/8-h dark cycle; the experiment was repeated three times. The number of germinated seedlings were recorded every 2 h from 34 to 96 h, until all the seeds achieved the maximum germination rate. The root length was measured and analyzed statistically, 7 days after germination.

The 7-day-old seedlings were stained using the nitroblue tetrazolium (NBT) method for detecting O_2_^-^ (Scarpeci et al., 2008; Kong et al., 2011). The seedlings were soaked in 0.5 mg/mL NBT solution for 20 h in dark and then boiled in 96% ethanol for 10 min to decolorize them. After cooling, the seedlings were placed in 60% glycerol and photographed.

### HPLC and HPLC-MS

Plant hormones, including ABA, SA, MeJA, and AZA, were extracted from fresh tobacco leaves and quantified by high-performance liquid chromatography-mass spectrometry (HPLC-MS) (Pan et al., 2010). For ABA, SA, and MeJA, the extraction procedures were as described, but for AZA, a different extraction solution (ethanol:water:: 80:20, vol/vol) was used. For quantification, an aliquot of plant hormone samples was analyzed by HPLC-MS (Aglient 1290 HPLC and SCIEX-6500 Qtrap), following the equipment setup as described (Pan et al., 2010). The separation of plant hormones was done by reverse-phase (RP-C_18_) HPLC by altering the elution gradient of solvent A (methanol/0.1% formic acid) and solvent B (water/0.1% formic acid), as per the following protocol: 0–2 min, A = 20%; 2–14 min, A increased linearly to 80%; 14–15 min, A maintained at 80%; 15.1, A decreased to 20%; 15.1–20 min, A maintained at 20%.

### Spectrophotometric Analysis

The reducing sugars were estimated by the 3,5-dinitrosalicylic acid (DNS) method (Wang, 2006). Glucose was dried at 80°C to a constant weight and used for preparing a standard curve. The content of reducing sugars was spectrophotometrically analyzed at 540 nm.

The total soluble sugars were measured by the anthrone method (Wang, 2006). Sucrose was dried at 80°C to a constant weight and used for preparing a standard curve. The content of total soluble sugars was analyzed spectrophotometrically at 630 nm.

Chlorophyll a and b were extracted with 95% ethanol; their absorbance was measured spectrophotometrically at 649 and 666 nm, and their content was determined following a previously described protocol (Wang, 2006).

The enzymatic activities of catalase (CAT) and peroxidase (POD) were also determined by spectrophotometric methods using kits (Nanjing Jiancheng Bioengineering Institute, China). The reactions for CAT and POD were performed at 37°C, and the optical densities were measured at 405 and 420 nm, respectively. The total protein concentration in the enzyme extract was quantified by Bicinchoninic acid (BCA) method using a total protein assay kit (No. A045-3, Nanjing Jiancheng Bioengineering Institute, China).

All the tests were conducted in triplicates for each of the three independent samples.

### Amplification of *NtGCN2*

The amplification of fragments and full length cDNAs of *NtGCN2-1* and *NtGCN2-2* was performed using routine PCR and 5′ and 3′ rapid amplification of cDNA ends (RACE), as described previously (Zhang et al., 2014). The PCR products were cloned into pMD19-T vector (Takara, Japan) and sequenced. The sequence of *NtGCN2* was assembled by DNA STAR (version 7.1.0) and used for bioinformatic analysis.

### Real-Time Quantitative RT-PCR

The transcription level was determined by real-time quantitative reverse transcription polymerase chain reaction (Real-time qRT-PCR) using a two-step reaction process. In each RT reaction, cDNA was synthesized from 0.5 μg total RNA using PrimerScript RT Master Mix (TaKaRa, Japan) in a GeneAmp^®^ PCR System 9700 (Applied Biosystems, United States), in following steps: reverse transcription at 37°C for 15 min, and inactivation of reverse transcriptase by heating at 85°C for 5 s. The product was then diluted 10-times in nuclease-free water and kept at -20°C.

Real-time PCR was carried out by LightCycler^®^ 480II Real-time PCR Instrument (Roche, Switzerland) using the following program: 95°C for 10 min, and 40 cycles of 95°C for 10 s, and 60°C for 30 s. Each sample was analyzed in triplicate. At the end of the PCR cycle, melting curve analysis was performed to validate the generation of the expected PCR product. The strategy for primer design was based on the alignment of cDNA sequences of *NtGCN2-1*, *NtGCN2-2*, *NsyGCN2*, and *NtoGCN2* (**Supplementary Figure S1**). The primer sequences that were used are shown in **Supplementary Table S1**. The expression levels of mRNAs were normalized to that of tobacco ribosome gene, *L25* (L18908), and were calculated using the 2^-ΔΔ*Ct*^ method (Livak and Schmittgen, 2001). The qRT–PCRs were performed in triplicate for each of the three independent samples.

### Vector Construction and Transformation of Tobacco With *NtGCN2*

Based on the obtained *NtGCN2-1* sequences, we designed the following primers for construction of the genetic transformation vector: GCN2-*Kpn*I-f (5′-GAAGGA GGTACCCCATGGGCAGCAGCCATCAT-3′, the underline indicates the *Kpn*I site) and GCN2-*Bam*HI-r (5′-TTGGTAGGATCCCTAGTTCCAGATGGATGGG-3′, the underline indicates the *Bam*HI site). The amplified *NtGCN2-1* was ligated into pC2300-OCS vector, which was then transformed into *Agrobacterium tumefaciens* (strain LBA4404). The transformed *A. tumefaciens* harboring the vector construct was subsequently used for generating stably transformed tobacco plants following the method reported previously (Sparkes et al., 2006).

The *NtGCN2* transformed plants were screened on solidified MS medium containing kanamycin (50 μg/mL) and were further identified by PCR. The confirmed transgenic plants were used for assessing the transcriptional level of *NtGCN2* by real-time qRT-PCR.

### Recombinant Protein Expression and Purification

The coding sequence for the kinase domain (KD) (1275–2208 nt) was amplified by high-fidelity PCR using gene-specific primers, and cloned into the expression vector, pET15b, forming pET15b-NtGCN2 (KD). After confirmation of the clone by sequencing, the vector construct was transformed into *Escherichia coli* strain BL21-CodonPlus-(DE3)-*RIPL*. The expression of recombinant protein was induced by 0.2 mM isopropyl β-D-1-thiogalactopyranoside (IPTG) at 16°C for 13 h. The bacterial cells were collected by centrifugation, resuspended in buffer (50 mM Tris-HCl, 50 mM NaCl, pH 8.0), and then lysed by sonication. The cell debris was removed by centrifugation at 10,000 × *g* for 20 min at 4°C, and the soluble target protein was collected in the supernatant. Subsequently, the proteins were subjected to purification procedures, including Ni^2+^-NTA (AKTA Prime100), anion ion exchange (Hitrap Q), and G0 molecular sieve, following the manufacturer’s instructions. The purified proteins were analyzed by sodium dodecyl sulfate polyacrylamide gel electrophoresis (SDS-PAGE). NteIF2α was cloned into pET15b and the resulting vector, pET15b-NteIF2α, was expressed in *E. coli* strain BL21-CodonPlus-(DE3)-*RIPL*; the recombinant protein was purified as described previously (Zhao et al., 2016; Chen et al., 2017).

### Immunoblot Analysis of eIF2α Phosphorylation

The phospho-eIF2α (S51) antibody (Catalog No. 9721, Cell Signaling, United States), generated in rabbit against human phosphorylated eIF2α, was used (at 1/1,000 dilution) for the detection of phosphorylated NteIF2α. After incubation with horseradish peroxidase-coupled anti-rabbit secondary antibody (Sigma 1/5,000 dilution), immunoblots were developed using the ECL Plus Western Blotting detection reagents. Chemiluminescence was visualized with a VersaDoc Imaging System (Bio-Rad Laboratories, United States). The equal loading of proteins was confirmed by reprobing the membranes with a plant-specific anti-β-actin (Sigma 1/5,000 dilution).

### GCN2 Activity Assay

The GCN2 activity assay was performed *in vitro*, as described previously (Berlanga et al., 1999). In brief, the reaction mixtures containing 20 mM Tris-HCl, pH 7.5, 0.2 mg/mL BSA, 10 μM ATP, 5 mM Mg(OAc)_2_, 2 μg purified NteIF2α substrate, 5 μCi of [γ-^32^P]ATP (3000 Ci mmol^-1^), and the indicated amounts of NtGCN2 (KD) were incubated at 30°C for 20 min. The reactions were terminated by adding 5 × SDS loading buffer (50 mM EDTA, 0.5% SDS, 25% glycerol, and 0.0025% bromophenol blue). The samples were electrophoresed on a 10% SDS-polyacrylamide gel and the gel was visualized using a phosphorimager and was scanned with Typhoon 9410 (GE Healthcare, Buchinghamshire, United Kingdom).

### Statistical Analysis

The data were analyzed by a simple variance analysis (SPSS version 17.0 for windows) and are presented as means ± standard error (SE) of three replicates. Statistical significance between the wild type (WT) and individual overexpression (OE) lines was determined by Student’s *t-* test at *P* < 0.05.

## Results

### Cloning of Two *NtGCN2s* From *N. tabacum*

Because *N. tabacum* is an allotetraploid crop in which different NtGCN2 genes could exist, we designed different primers for PCR and finally obtained two different cDNA sequences. The two products were cloned into pMD-18 vector. These genes were named *NtGCN2-1* and *NtGCN2-2*; they had the same size (3,759 bp), but their sequence varied slightly in the coding region. The sequences of *NtGCN2-1* and *NtGCN2-2* were submitted to the NCBI database under accession numbers KJ706220 and KR184727, respectively.

The open reading frames (ORFs) of *NtGCN2-1* and *NtGCN2-2* encoded proteins containing 1252 amino acids, with a high sequence similarity (97% identity). These proteins contained the typical protein kinase catalytic domain and the RWD domain, which are conserved in the eIF2α kinase, GCN2. In addition, BLASTP alignment showed that NtGCN2 was highly conserved not only in the two wild tobacco species *N. sylvestris* and *N. tomentosiformis*, but also in GCN2 of *Solanum tuberosum* (90% identity) and *Solanum lycopersicum* (88% identity) (**Supplementary Figure S2**).

The results of phylogenetic analysis (**Supplementary Figure S3**) demonstrated that the amino acid sequence of NtGCN2-1 was close to that of NtoGCN2, whereas NtGCN2-2 showed higher similarity to NsyGCN2. The two *Solanum* proteins, StGCN2 and SlyGCN2, were also classified together with *Nicotiana* GCN2. Interestingly, the sequences of two GCN2s, TaGCN2 and ObGCN2, from monocots, were more similar to that of *Nicotiana* GCN2 than to those of GCN2s from other dicots (**Supplementary Figure S3**). AtGCN2, previously reported by Zhang et al. (2003), together with EsGCN2, CrGCN2, and AlyGCN2, belonged to the same clade. GCN2s from *fabids*, *Vitales*, and *malvids* were classified into different clades, which were far from that of the *Solanales*, including NtGCN2.

### Expression of *NtGCN2* Is Induced by Different Treatments

**FIGURE 1:**
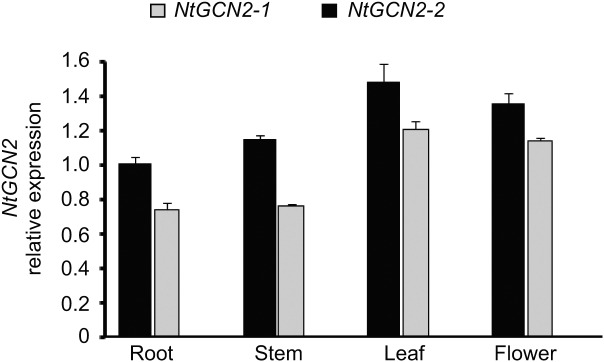
Relative transcriptional level of *NtGCN2-1* and *NtGCN2-2* in different organs of *Nicotiana tabacum* K326. Total RNA was isolated from different organs of *N. tabacum* K326. Transcriptional levels of *NtGCN2-1* (gray) and *NtGCN2-2* (black) were detected by real-time qRT-PCR. Data were normalized by comparing with *NtGCN2-2* in the root. Each test item was conducted in triplicates for each of the three independent samples. Error bars indicate the standard error.

The effect of different treatments on *NtGCN2* expression was analyzed by real-time qRT-PCR. Firstly, we analyzed the transcriptional level of *NtGCN2* in different organs without the treatments. As shown in **Figure 1**, the *NtGCN2* transcript levels varied in the roots, stems, leaves, and flowers of tobacco. Both *NtGCN2-1* and *NtGCN2-2* showed similar expression patterns in the organs: the highest transcription level was present in the leaves, and the lowest level was present in the roots. *NtGCN2-2* was observed to have a higher expression level than *NtGCN2-1* in the tested organs.

**FIGURE 2:**
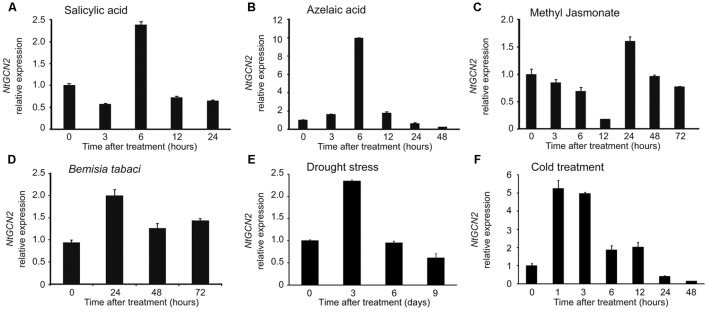
Analysis of relative expression level of *NtGCN2* after exposure to different treatments. Total RNA was isolated from tobacco plants treated by salicylic acid **(A)**, azelaic acid **(B)**, methyl jasmonate **(C)**, *B. tabaci* infection **(D)**, drought **(E)**, and cold **(F)**. After cDNA synthesis, relative expression level of *NtGCN2* was determined by real time qRT-PCR using primers GCN2-F and GCN2-R. Each test item was conducted in triplicates for each of the three independent samples. Error bars indicate the standard error.

To investigate the response of *NtGCN2* expression to different treatments, the transcriptional levels of *NtGCN2*, including those of both *NtGCN2-1* and *NtGCN2-2*, were analyzed by qRT-PCR after exposure to SA, AZA, MeJA, drought, cold, and *B. tabaci* infection. As shown in **Figures 2A,B**, the expression of *NtGCN2* was induced by SA and AZA in a similar pattern. The transcription of *NtGCN2* increased after the addition of the two hormones, and reached the maximum level at 6 h, after which it decreased. After 6 h of the treatment, the transcriptional level of *NtGCN2* was induced 2.5-fold by SA, whereas it increased by 10.3-fold upon AZA treatment. However, the transcription of *NtGCN2* after MeJA treatment decreased continuously between 3 to 12 h; it was then stabilized until increasing sharply at 24 h and then declined gradually (**Figure 2C**). Compared to these phytohormone treatments, the transcription of *NtGCN2* upon *B. tabaci* infection showed a pattern similar to that observed under the SA treatment. The transcription level reached its maximum at 24 h, and was then decreased but maintained at a level higher than that present in the beginning (**Figure 2D**). The transcript level of *NtGCN2* reached its peak after drought treatment for 3 days and after 1 h of cold treatment (**Figures 2E,F**).

### NteIF2α Phosphorylation Is Induced Under Different Treatments

**FIGURE 3:**
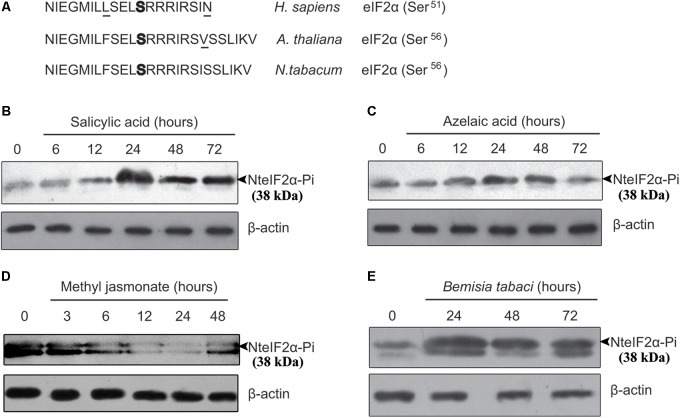
Analysis of phosphorylation of NteIF2α under different stress treatments. **(A)** Amino acid sequence alignment of eIF2α from *H. sapiens*, *A. thaliana* and *N. tabacum*. The phosphorylation site of serine was highlighted in bold. The amino acid residues not identical were underlined. **(B–E)** Time-course of NteIF2α phosphorylation in response to treatment of salicylic acid **(B)**, azelaic acid **(C)**, methyl jasmonate **(D)**, and *B. tabaci* infection **(E)**. NteIF2α phosphorylation was determined by an antibody that specifically recognizes the phosphorylated form of NteIF2α (upper panel). Total proteins in each lane were demonstrated by β-actin recognized by a specific antibody (lower panel).

To detect the phosphorylation of NteIF2α, a specific antibody for detecting the phosphorylated form of NteIF2α was selected. We compared the eIF2α sequences of *Homo sapiens*, *A. thaliana*, and *N. tabacum*, and found that the amino acid sequence surrounding the phosphorylation site of serine shared the same core sequence E-L-S(p)-R-R (**Figure 3A**). Therefore, the same CST polyclonal antibody could be used for the detection of phosphorylated NteIF2α in western blot analysis of NteIF2α phosphorylation.

The NteIF2α phosphorylation shown in **Figure 3** was altered upon treatment with any of the plant hormones (SA, AZA, and MeJA) or under biotic stress (*B. tabaci* infection). During SA treatment, the phosphorylation of NteIF2α was activated gradually and peaked at 24 h; the phosphorylation was then decreased but remained stable at a high level until 72 h (**Figure 3B**). Compared to the SA treatment, the phosphorylation of NteIF2α in response to AZA showed a similar pattern: the phosphorylation peaked at 24 h, and declined thereafter (**Figure 3C**). The *B. tabaci* infection also induced the phosphorylation of NteIF2α, which strongly increased at 24 h and then gradually decreased at 48 and 72 h (**Figure 3E**). The MeJA treatment, however, led to a different response of NteIF2α phosphorylation. The level of NteIF2α phosphorylation was first decreased, and then appeared to recover at 48 h after the treatment (**Figure 3D**).

### NtGCN2 Can Phosphorylate NteIF2α *in Vivo* and *in Vitro*

**FIGURE 4:**
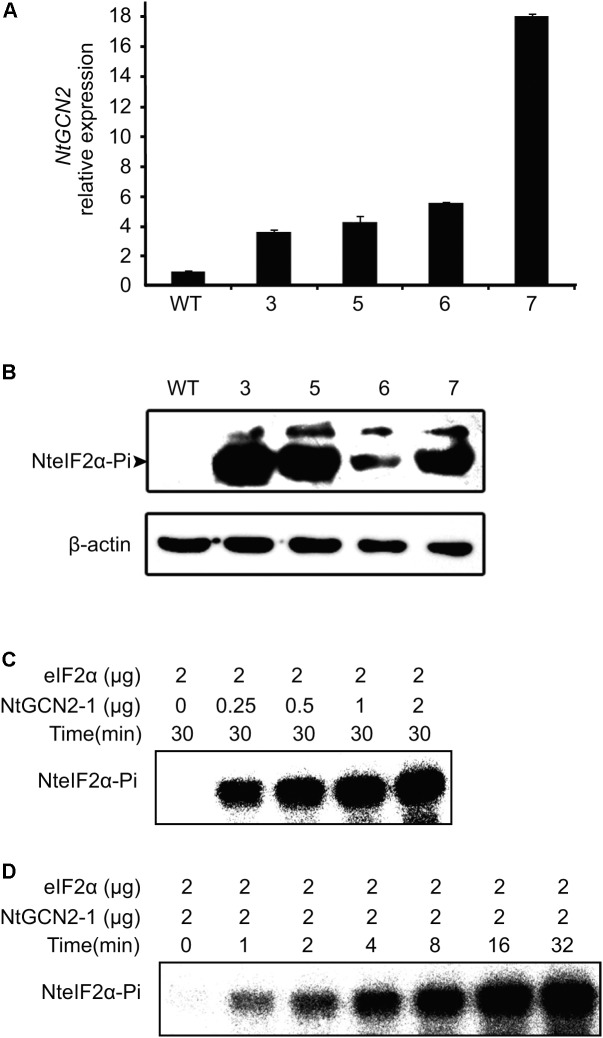
Characterization of NteIF2α phosphorylation by NtGCN2 *in vivo* or *in vitro.* **(A)** Real time qRT-PCR analysis of *NtGCN2-1* overexpression (OE) plants. Total RNA was isolated from leaves and used for cDNA synthesis and real time qRT-PCR. Three samples were taken from each plant. WT, wild type of *N. tabacum* K326; line 3, 5, 6 and 7, OE plants. **(B)** Western-blotting analysis of the phosphorylation of NteIF2α *in vivo*. NteIF2α phosphorylation was determined by an antibody that specifically recognizes the phosphorylated form of NteIF2α (upper panel). Total proteins in each lane were demonstrated by β-actin recognized by a specific antibody (lower panel). WT, wild type of *N. tabacum* K326; 3, 5, 6, and 7, OE plants. **(C,D)** *In vitro* assay of protein kinase activity of recombinant NtGCN2-1 protein. Different amount of NtGCN2-1 **(C)** and different incubation time **(D)** were adopted for the assay.

According to the above results, stress conditions can induce both *NtGCN2* expression and NteIF2α phosphorylation. However, the causality between two results is uncertain. To this end, we generated transgenic *N. tabacum* K326 plants overexpressing *NtGCN2-1* by *Agrobacterium*-mediated transformation with a pC2300-OCS-*NtGCN2-1* vector, which contained kanamycin resistance gene as a selection marker. Five positive lines were obtained by kanamycin selection and four lines were identified by PCR using gene- and vector-specific primers (**Supplementary Figure S4**). Real time qRT-PCR showed that the transcriptional level of *NtGCN2* in these transformed plants was obviously higher than that in WT plant. Noticeably, the *NtGCN2* expression in line 7 was 17-fold higher compared to the expression in WT (**Figure 4A**). The levels of phosphorylated NteIF2α in the OE lines were much higher than that in WT plant, correlating with the *NtGCN2* transcript level, indicating that NtGCN2 was involved in the phosphorylation of NteIF2 *in vivo* (**Figure 4B**). Furthermore, recombinant NtGCN2-1 could also phosphorylate eIF2α *in vitro* (**Figures 4C,D**): eIF2α phosphorylation only happened when protein was added in the reaction, and phosphorylation was increased with the increasing amount of NtGCN2-1 and with the incubation time.

### Overexpression of *NtGCN2-1* Promotes Seed Germination and Increases Root Length

**FIGURE 5:**
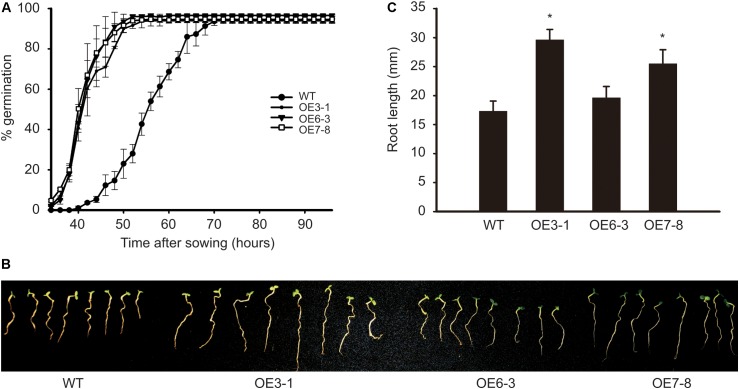
Determination of OE seed germination and seedling growth. WT and OE seeds germinated on filter paper. The percentage of germination were calculated in every 2 h **(A)**. The photography **(B)** and root length measurement **(C)** were done at 7 days after sowing. Each test item was detected in triplicate for each of the three independent samples. Error bars indicate the standard error. Asterisks indicated statistical significance between WT and individual OE line determined by student’s *t*-test (^∗^*P* < 0.05).

The OE plants mentioned above produced progenies by self-pollination. Three plants, namely OE3-1, OE6-3, and OE7-8 derived from lines 3, 6, and 7, respectively, were identified as homozygous T-DNA insertion lines. The seeds and seedlings of these OE lines were used for the following analysis. The activity of GCN2 in *Arabidopsis* was shown to negatively regulate the germination of seeds under diverse environmental conditions (Liu et al., 2015). Here, we studied the influence of *NtGCN2* on seed germination and seedling growth in the OE lines. The WT and OE seeds were germinated on filter papers soaked in MS medium in a growth chamber. The germination of seeds was first observed in the OE lines at 34 h, whereas the WT seeds germinated at 40 h. The percentage of germination for OE lines increased dramatically, and it took about 20 h (at 54 h) for the three OE lines to reach the maximum germination rate, which was faster than that for the WT seeds, which took 32 h more to achieve the maximum germination rate 72 h. These results implied that the overexpression of *NtGCN2-1* accelerates seed germination (**Figure 5A**). Besides, *NtGCN2-1* overexpression exhibited a positive effect on the seedling morphology. After growing for 7 days, two OE seedlings (OE3-1 and OE7-8) were observed to have a bigger size than the WT seeds (**Figure 5B**). To quantify the difference, the root lengths of the OE and WT seedlings were measured and the results showed that the roots of OE seedlings were longer than those of WT seedlings (**Figure 5C**).

### Overexpression of *NtGCN2-1* Increases the Antioxidant Capacity

**FIGURE 6:**
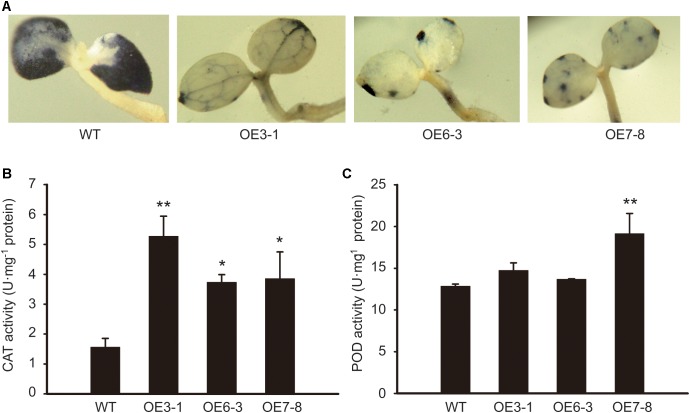
Comparison of antioxidant capacity between WT and OE plants. **(A)** 7-day-old seedlings of OE and WT were stained by NBT and photographed. NBT, nitroblue tetrazolium. **(B,C)** The CAT and POD activities were measured using young leaves. CAT, catalase; POD, guaiacol peroxidase. Each test item was detected in triplicate for each of the three independent samples. Error bars indicate the standard error. Asterisks indicated statistical significance between WT and individual OE line determined by student’s *t*-test (^∗^*P* < 0.05, ^∗∗^*P* < 0.01).

To investigate whether NtGCN2 plays a role in the regulation of antioxidant system, we determined the antioxidant capacity of tobacco OE plants by NBT staining, and measured the CAT and POD activities. The NBT infiltration can be used for the visualization of the superoxide anion (O_2_^-^), which is the major oxidant species responsible for reducing NBT to form an insoluble formazan. The results showed that the dark blue insoluble formazan compound accumulated in the WT seedlings, spreading almost all over the cotyledons (**Figure 6A**). In contrast, in the cotyledons of the OE lines, the insoluble compound was distributed only in some veins, or showed a dotted pattern near the leaf edge, suggesting that the O_2_^-^ accumulation in the cotyledons of WT was much higher than that in the OE lines. Correspondingly, the activity of CAT in the OE lines was significantly higher than that in the WT plants (**Figure 6B**). The results of POD were not so obvious, but a higher activity was observed in the OE3-1 and OE7-8 lines (**Figure 6C**).

### Overexpression of *NtGCN2-1* Changes the Contents of Endogenous Hormones

**FIGURE 7:**
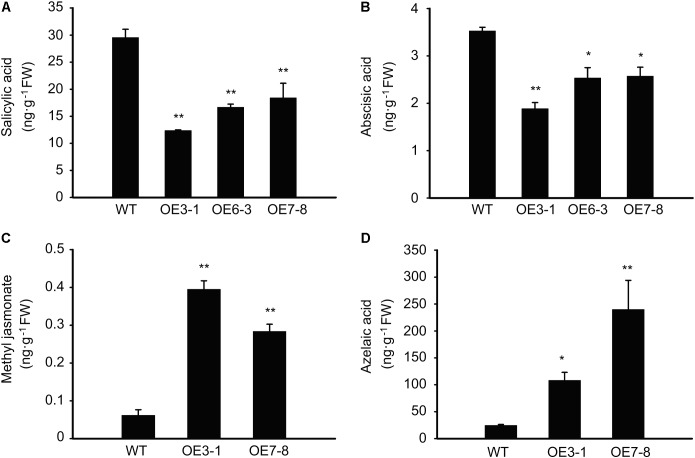
Content of the endogenous phytohormones in the WT and OE plants. **(A–D)** HPCL analysis for content of phytohormones including salicylic acid, methyl jasmonate, abscisic acid, and azelaic acid. Asterisks indicated statistical significance between the WT and the tested OE lines determined by student’s *t*-test (^∗^*P* < 0.05, ^∗∗^*P* < 0.01).

As revealed by the above-mentioned results, external plant hormones, such as SA, MeJA, and AZA, have an impact on the expression of *NtGCN2*. Therefore, we also wanted to know the effect of *NtGCN2* overexpression on the endogenous phytohormones. The results shown in **Figure 7** revealed that two trends existed for these phytohormones in the OE plants. The contents of SA and ABA were significantly decreased in the OE lines, whereas those of MeJA and AZA were dramatically increased in the tested OE lines.

### Overexpression of *NtGCN2-1* Alters the Contents of Sugar and Chlorophyll

**FIGURE 8:**
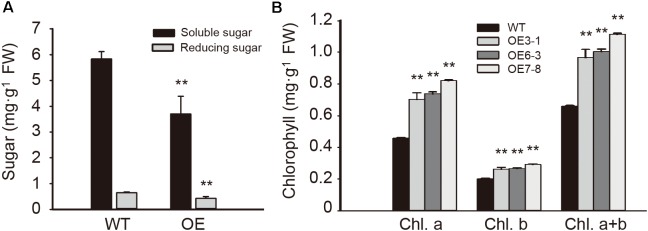
Content of sugar and chlorophyll in the WT and OE leaves. **(A)** Content of soluble sugar (black) and reducing sugar (gray) in wild type (WT) and overexpression (OE) plants. **(B)** Content of chlorophyll in WT and OE plants. Each test item was detected in triplicate for each of the three independent samples. Error bars indicate the standard error. Asterisks indicate statistical significance between WT and OE lines determined by student’s *t*-test (^∗∗^*P* < 0.01).

To examine the influence of *NtGCN2-1* overexpression on plant metabolism, the contents of sugars and chlorophyll were measured and are shown in **Figure 8**. Compared to the WT plants, the contents of total soluble sugars and reducing sugars in the OE plants were considerably lower. In contrast, different results were obtained for the chlorophyll content. The contents of chlorophyll a and b in the OE plants were significant higher than in the WT plants, and therefore, the OE plants had a higher content of total chlorophyll compared to the WT plants.

## Discussion

We cloned two *NtGCN2s*, namely *NtGCN2-1* and *NtGCN2-2*, from *N. tabacum* K326; both these genes encoded proteins of 1252 amino acid residues and showed only a slight difference in their sequence (**Supplementary Figure S2**). The protein sequences included a typical eIF2α kinase and a RWD domain. The alignment of amino acid sequences showed that the two NtGCN2s of *N. tabacum* were closely related to NsyGCN2 and NtoGCN2, respectively, in agreement with the fact that *N. sylvestris* and *N. tomentosiformis* are the maternal and paternal genome donors of tobacco, respectively (Skalicka et al., 2005; Yukawa et al., 2006). The GCN2s of monocots and dicots were not separated in the phylogenetic tree, indicating that GCN2 is conserved and has not diverged in monocots and dicots. Many higher plants contain only one GCN2 gene, whereas tobacco exhibits gene redundancy because of its allotetraploidy.

Generally, GCN2 can be activated and can phosphorylate eIF2α under conditions of amino acid and purine depletion in yeast and *Arabidopsis* after wounding, or after exposure to UV, MeJA, and SA (Hinnebusch, 1997; Goossens et al., 2001; Lageix et al., 2008). In the present study, we also found that *NtGCN2* was induced at the transcriptional level and the amount of its catalytic product, eIF2α-Pi, was increased under SA, AZA, MeJA treatments, and *B. tabaci* infection (**Figures 2**, **3**). Noticeably, the patterns of transcription and product alteration were similar: peak shaped for SA, AZA, and *B. tabaci* infection, and down- to up-regulation for MeJA. Furthermore, the change in expression occurred earlier than phosphorylation. These results indicated that *NtGCN2* expression was closely related to eIF2α phosphorylation under stress conditions. The *in vivo* and *in vitro* assays confirmed the phosphorylation of eIF2α by NtGCN2 (**Figure 4**). These results also indicate that NtGCN2 is involved in the biotic and abiotic stress response in tobacco.

Our data demonstrated that seed germination was accelerated by *NtGCN2* overexpression. Besides, the growth of roots was promoted in the *NtGCN2* OE lines as well (**Figure 5**). In comparison, previous studies showed that seed germination was delayed by GCN2 deficiency in the *Arabidopsis atgcn2* mutants (Liu et al., 2015). Similar results in this study indicated that the acceleration of seed germination and longer roots occurred in these OE lines along with ABA reduction. This could be explained by the evidence that high concentration of ABA inhibits seed germination and root growth by promoting ethylene biosynthesis (Debeaujon and Koornneef, 2000; Millar et al., 2006; Luo et al., 2014). Taken together, these results indicate that *NtGCN2* exerts positive effects on seed germination and root growth through the suppression of the ABA level. Additionally, ABA induced the accumulation of total soluble sugars and reducing sugars in tobacco cells (LaRosa et al., 1987). In the *NtGCN2* OE plants, the ABA content was decreased, and correspondingly the contents of total soluble sugars and reducing sugars also decreased, indicating that *NtGCN2* overexpression shift the plants to a stress-responsive status by reducing the nutrient levels through ABA.

It is well known that GCN2 is a sensor for amino acid depletion and responds to dietary nutrients in yeast and mammals. It possible plays a role in plant nutrition-related physiological changes during the growth and development in *Arabidopsis* (Liu et al., 2015). In plants, the contents of chlorophyll a and b are considered as indicators of the nutrient status. The total content of chlorophyll increased in the *gcn2* mutants with loss of AtGCN2 function (Liu et al., 2015). In contrast, a recent study showed that the *gcn2* mutants contained less chlorophyll and showed delayed growth than the WT plants with the activation of GCN2-dependent eIF2α phosphorylation after sulfonylurea treatment (Zhao et al., 2018), whereas the chlorophyll content showed no significant difference under the normal condition (Liu et al., 2015). Furthermore, higher contents of chlorophyll a and b were detected in the *NtGCN2* OE lines, which were also shown to have relatively higher levels of eIF2α phosphorylation (**Figure 6**). These results suggested that GCN2 functions in the maintenance of chlorophyll content through eIF2α phosphorylation.

Plant hormones have been shown to play important roles during plant defense response and confer disease resistance in plants (Bari and Jones, 2009). In our study, the phytohormones including SA, AZA, and MeJA, were applied to tobacco plants. The results showed that these hormones could act as stress stimuli and induce the expression of *NtGCN2*. In turn, *NtGCN2* also affected the contents of endogenous hormones. SA and MeJA are endogenous signaling molecules for tissue injury in plants, participating in the activation of GCN2-dependent eIF2α phosphorylation and generate stress response to insect herbivores (Lageix et al., 2008). However, different trends were observed for SA and MeJA with regard to the induction of gene expression (**Figure 2**), and the contents of endogenous hormones in the OE lines (**Figure 7**), indicating that in tobacco GCN2 activates the JA pathway and represses the SA signaling pathway to respond to biotic or abiotic stresses.

Azelaic acid has been identified as a pathogen-induced mobile metabolite, which can prime the plants to accumulate SA and participates in local- and long-distance resistance (Jung et al., 2009; Parker, 2009; Chanda et al., 2011; Chaturvedi et al., 2012). In the present study, SA and AZA were observed to activate the expression of *GCN2* and induced the phosphorylation of NteIF2α. AZA was accumulated when *NtGCN2* expression was up-regulated, whereas SA was reduced in the OE lines. Taken together, these results indicate that the accumulation of AZA might prime the plants to respond to stresses whereas different levels of AZA and SA are required for the GCN2-mediated stress response.

Salicylic acid promotes the production of peroxides and inhibits the peroxide-scavenging activity of antioxidant enzymes; thus, an increase in the SA level facilitates the oxidative burst (Rao et al., 1997). In this study, the lower SA levels in the OE plants correlated with the lesser peroxide accumulation and higher activities of CAT and POD (**Figure 6**), indicating that the higher antioxidant activity in OE plants might be triggered through SA repression.

In summary, NtGCN2 catalyzes the phosphorylation of NteIF2α. The overexpression of *NtGCN2* not only results in the accumulation of phosphorylated NteIF2α, but also changes the physiological status of plants that improves their stress tolerance.

## Author Contributions

NL conducted the experiments and wrote the manuscript. S-jZ, QZ, YL, and HG prepared samples and figures. H-fJ, Y-xY, and H-yZ discussed the data. X-fY tested the content of chlorophyll. S-tZ designed the experiments and wrote the manuscript.

## Conflict of Interest Statement

The authors declare that the research was conducted in the absence of any commercial or financial relationships that could be construed as a potential conflict of interest.

## Supplementary Material

FIGURE S1 Primer design for real time qRT-PCR of *NtGCN2*. Sequence alignment was done for the 5′ untranslated region (5′UTR) and partial open reading frame (ORF) of cDNA sequences of *NtGCN2-1*, *NtGCN2-2*, *NsyGCN2*, and *NtoGCN2*, to find the difference among these sequences. The identical nucleotides were highlighted in black, and the absent positions are represented by dashes. Position and direction of each primer are demonstrated by arrows. The different nucleotides at the end of some primers were pointed out by letter.

FIGURE S2 Amino acid sequence alignment of GCN2 from *N. tabacum* and some *Solanaceae* plants. Amino acid sequence alignment of GCN2 were done with Clustal X2.1. Identical amino acid residues are highlighted in black, and distinct residues are shown in gray. The gaps indicate the relevant residues are not present in the proteins. The conserved domains of RWD and protein kinase are underlined by solid line and dash line, respectively.

FIGURE S3 Phylogenetic analysis of putative GCN2 proteins from different plants. Proteins from different plants were matched by a protein BLAST search in plants using NtGCN2-1 as quarry sequence, and are shown in this figure, including NtGCN2-1 and NtGCN2-2, *Nicotiana tabacum* GCN2-1 and GCN2-2 (this work); NsyGCN2, *Nicotiana sylvestris* GCN2; NtoGCN2, *Nicotiana tomentosiformis* GCN2; SlyGCN2, *Solanum lycopersicum* GCN2 (XP_004250832.1); StGCN2, *Solanum tuberosum* GCN2 (XP_006352074.1); SiGCN2, *Sesamum indicum* GCN2 (XP_011101601.1); ObGCN2, *Oryza brachyantha* GCN2 (XM_006652390); TaGCN2, *Triticum aestivum* GCN2 (FR839672); AtGCN2, *Arabidopsis thaliana* GCN2 (AEE79918.1); AlyGCN2, *Arabidopsis lyrata subsp. lyrata* GCN2 (XP_002876502.1); CrGCN2, *Capsella rubella* GCN2 (XP_006292311.1); EsGCN2, *Eutrema salsugineum* GCN2 (XP_006402691.1); GmGCN2, *Glycine max* GCN2 (XP_006592149.1); PvGCN2, *Phaseolus vulgaris* GCN2 (XP_007131951.1); CaGCN2, *Cicer arietinum* GCN2 (XP_004507335.1); CsaGCN2, *Cucumis sativus* GCN2 (XP_004140982.1); VvGCN2, *Vitis vinifera* GCN2 (XP_002264839.2); FvGCN2, *Fragaria vesca* GCN2 (XP_004309842.1); TcGCN2, *Theobroma cacao* GCN2 (XP_007033960.1); CsiGCN2, *Citrus sinensis* GCN2 (XP_006478695.1); GrGCN2, *Gossypium raimondii* GCN2 (XP_012454129.1); PtGCN2, *Populus trichocarpa* GCN2 (XP_002310436.2); PeGCN2, *Populus euphratica* GCN2 (XP_011012928.1); RcGCN2, *Ricinus communis* GCN2 (XP_002533444.1). The sequence alignment was performed by Clustal W 1.83 program, and the phylogenetic tree was constructed by Phylip program using the neighbor-joining method. Bootstrap value was 100.

FIGURE S4 Identify *NtGCN2-1*-transformed plants by PCR. P, positive control, recombination vector of pC2300-OCS-*NtGCN2*-1 was used as positive control; WT, wild type K326 plant was used as negative control; 3 – 7, five transformed lines were tested.

TABLE S1 Primers for real time qRT-PCR analysis of *GCN2* in *Nicotiana tabacum.*
