# Supplementary figures and images for: Overexpression of Tobacco GCN2 Stimulates Multiple Physiological Changes Associated With Stress Tolerance
Source: Front Plant Sci. 2018 Jun 1;9:725. doi: 10.3389/fpls.2018.00725 (PMC5992440; doi:10.3389/fpls.2018.00725)

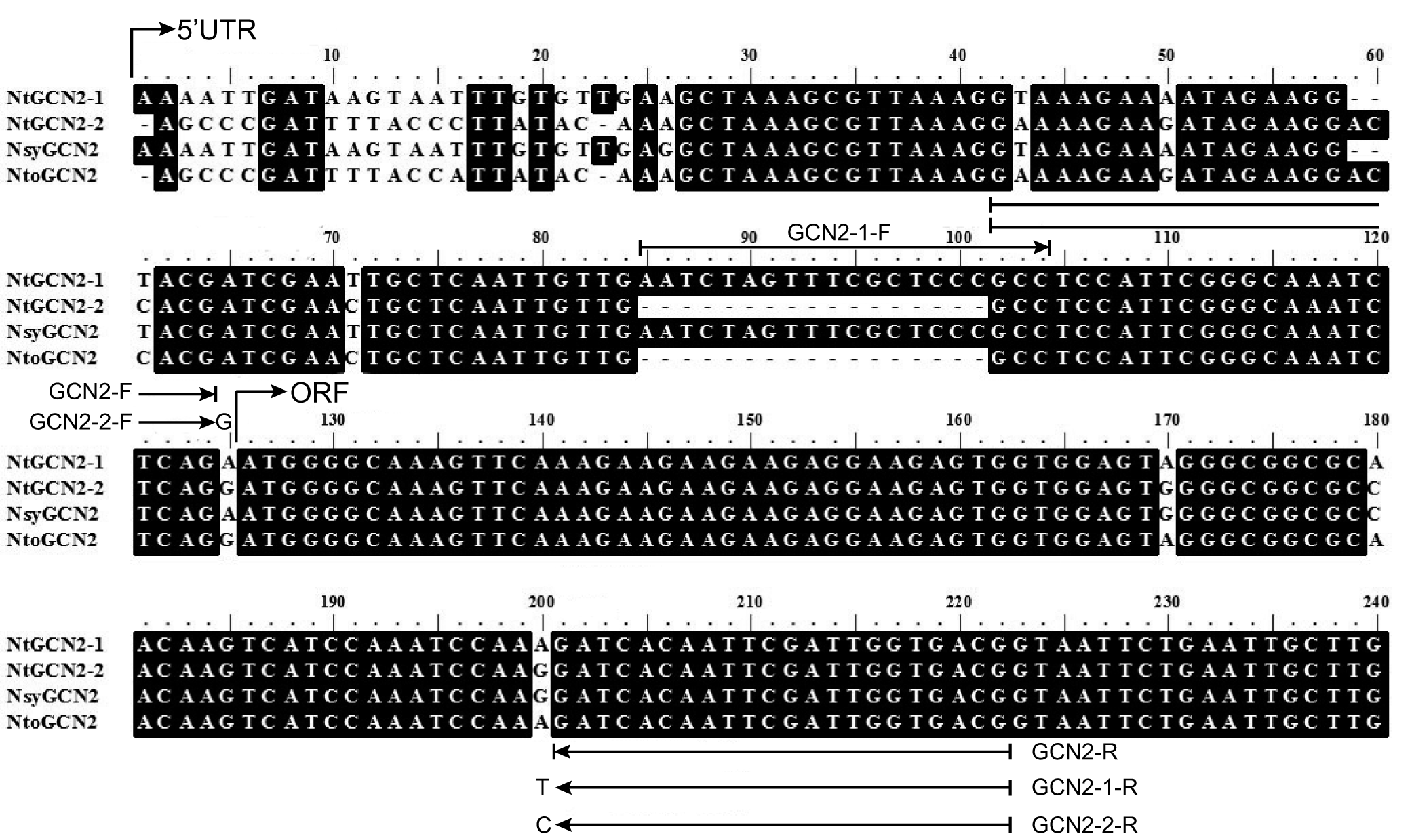

Supplement: FIGURE S1 — Primer design for real time qRT-PCR of NtGCN2. Sequence alignment was done for the 5′ untranslated region (5′UTR) and partial open reading frame (ORF) of cDNA sequences of NtGCN2-1, NtGCN2-2, NsyGCN2, and NtoGCN2, to find the difference among these sequences. The identical nucleotides were highlighted in black, and the absent positions are represented by dashes. Position and direction of each primer are demonstrated by arrows. The different nucleotides at the end of some primers were pointed out by letter. [file Image_1.TIF]

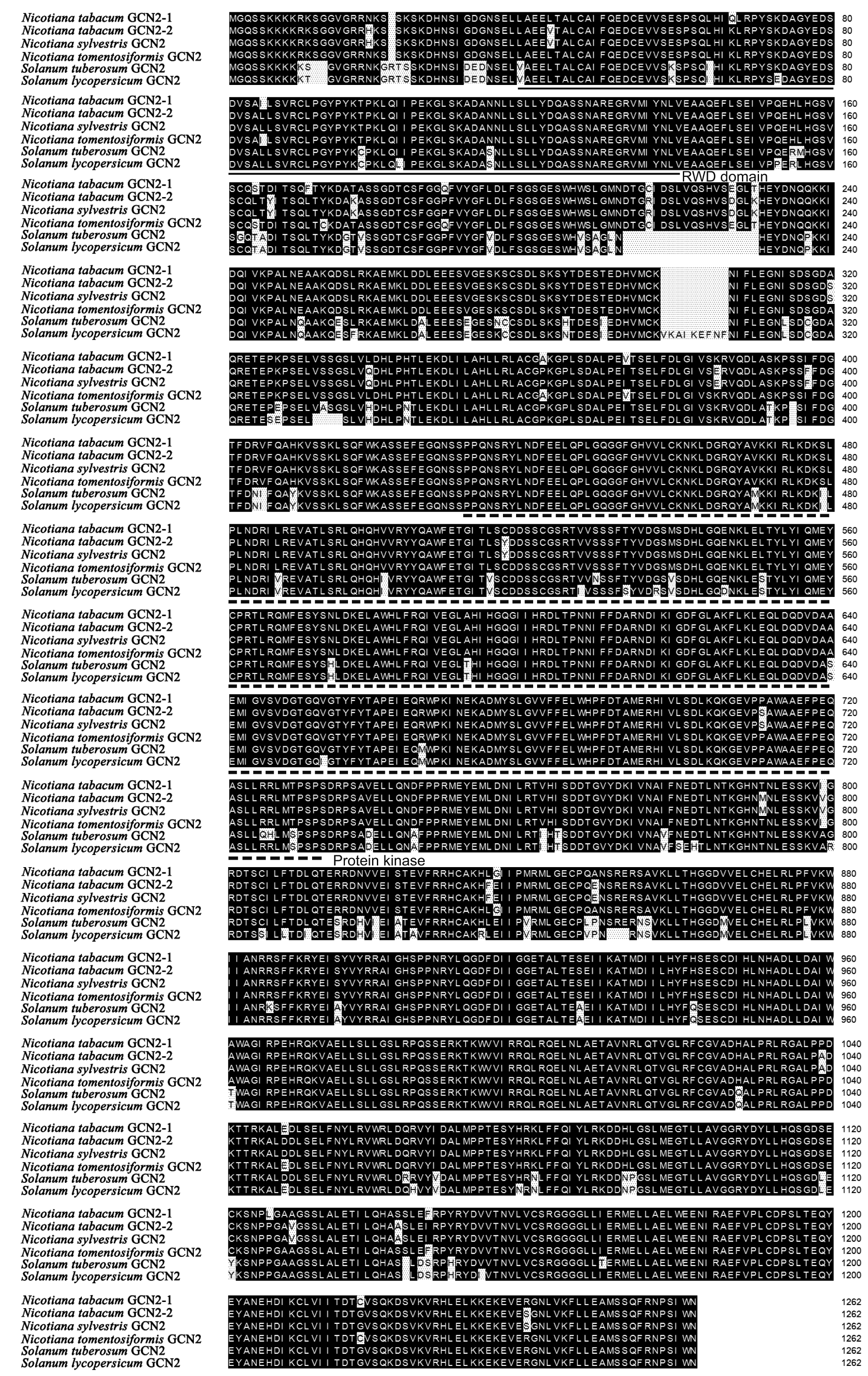

Supplement: FIGURE S2 — Amino acid sequence alignment of GCN2 from N. tabacum and some Solanaceae plants. Amino acid sequence alignment of GCN2 were done with Clustal X2.1. Identical amino acid residues are highlighted in black, and distinct residues are shown in gray. The gaps indicate the relevant residues are not present in the proteins. The conserved domains of RWD and protein kinase are underlined by solid line and dash line, respectively. [file Image_2.TIF]

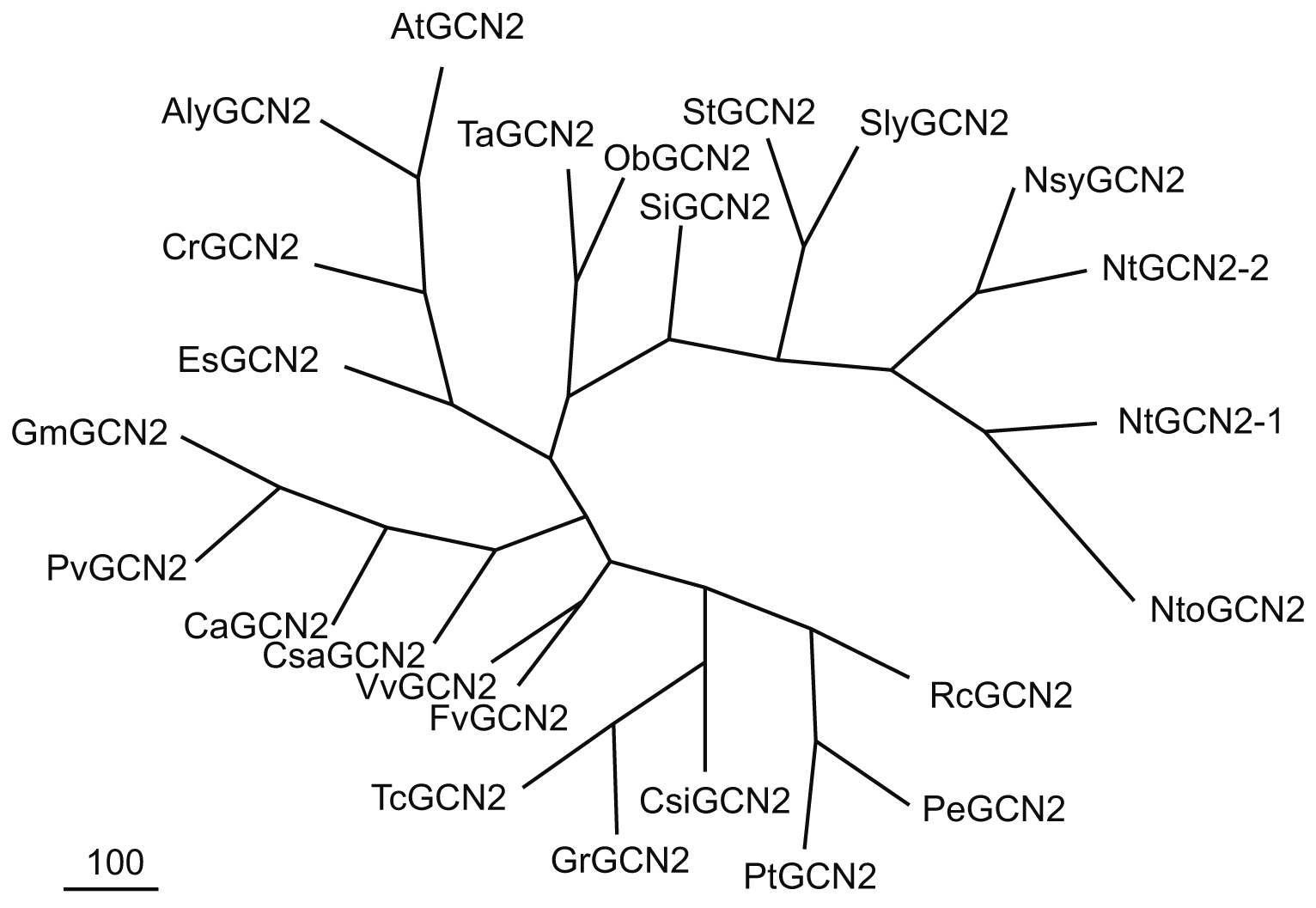

Supplement: FIGURE S3 — Phylogenetic analysis of putative GCN2 proteins from different plants. Proteins from different plants were matched by a protein BLAST search in plants using NtGCN2-1 as quarry sequence, and are shown in this figure, including NtGCN2-1 and NtGCN2-2, Nicotiana tabacum GCN2-1 and GCN2-2 (this work); NsyGCN2, Nicotiana sylvestris GCN2; NtoGCN2, Nicotiana tomentosiformis GCN2; SlyGCN2, Solanum lycopersicum GCN2 (XP_004250832.1); StGCN2, Solanum tuberosum GCN2 (XP_006352074.1); SiGCN2, Sesamum indicum GCN2 (XP_011101601.1); ObGCN2, Oryza brachyantha GCN2 (XM_006652390); TaGCN2, Triticum aestivum GCN2 (FR839672); AtGCN2, Arabidopsis thaliana GCN2 (AEE79918.1); AlyGCN2, Arabidopsis lyrata subsp. lyrata GCN2 (XP_002876502.1); CrGCN2, Capsella rubella GCN2 (XP_006292311.1); EsGCN2, Eutrema salsugineum GCN2 (XP_006402691.1); GmGCN2, Glycine max GCN2 (XP_006592149.1); PvGCN2, Phaseolus vulgaris GCN2 (XP_007131951.1); CaGCN2, Cicer arietinum GCN2 (XP_004507335.1); CsaGCN2, Cucumis sativus GCN2 (XP_004140982.1); VvGCN2, Vitis vinifera GCN2 (XP_002264839.2); FvGCN2, Fragaria vesca GCN2 (XP_004309842.1); TcGCN2, Theobroma cacao GCN2 (XP_007033960.1); CsiGCN2, Citrus sinensis GCN2 (XP_006478695.1); GrGCN2, Gossypium raimondii GCN2 (XP_012454129.1); PtGCN2, Populus trichocarpa GCN2 (XP_002310436.2); PeGCN2, Populus euphratica GCN2 (XP_011012928.1); RcGCN2, Ricinus communis GCN2 (XP_002533444.1). The sequence alignment was performed by Clustal W 1.83 program, and the phylogenetic tree was constructed by Phylip program using the neighbor-joining method. Bootstrap value was 100. [file Image_3.TIF]

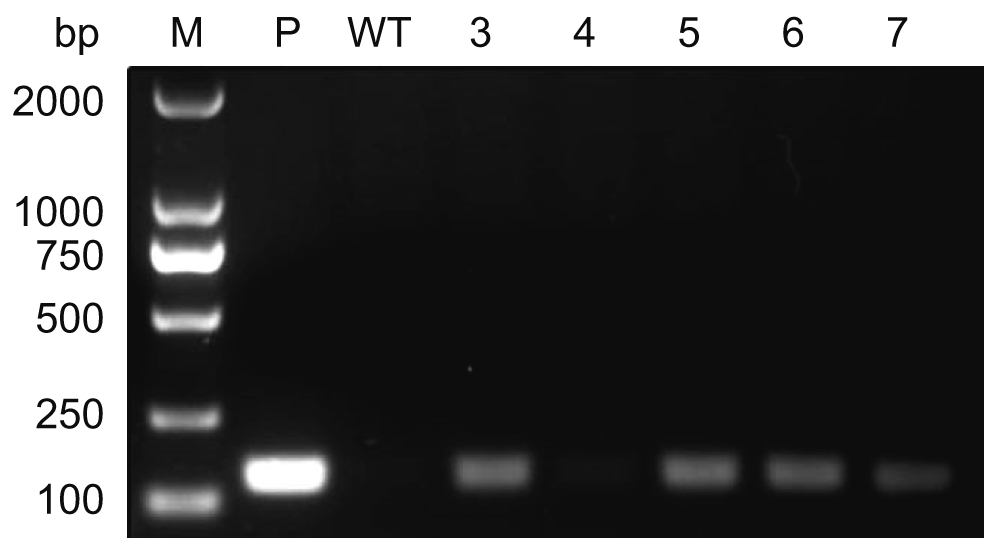

Supplement: FIGURE S4 — Identify NtGCN2-1-transformed plants by PCR. P, positive control, recombination vector of pC2300-OCS-NtGCN2-1 was used as positive control; WT, wild type K326 plant was used as negative control; 3 – 7, five transformed lines were tested. [file Image_4.TIF]
