# Supplementary material for: Overexpression of Tobacco GCN2 Stimulates Multiple Physiological Changes Associated With Stress Tolerance
Source: Front Plant Sci. 2018 Jun 1;9:725. doi: 10.3389/fpls.2018.00725 (PMC5992440; doi:10.3389/fpls.2018.00725)
Supplement: TABLE S1 — Primers for real time qRT-PCR analysis of GCN2 in Nicotiana tabacum. [file Table_1.DOCX]

Supplemental Table S1

Table S1 Primers for real time qRT-PCR analysis of *GCN2* in *Nicotiana tabacum*

| Primer | Sequence (5'-3') | Fragment size (bp) | Purpose |
| --- | --- | --- | --- |
| GCN2-1-F: | AATCTAGTTTCGCTCCCGCC | 138 | Amplification of *NtGCN2-1* |
| GCN2-1-R: | CGTCACCAATCGAATTGTGATCT |  |  |
| GCN2-2-F: | GCCTCCATTCGGGCAAATCTCAGG | 121 | Amplification of *NtGCN2-2* |
| GCN2-2-R: | CGTCACCAATCGAATTGTGATCC |  |  |
| GCN2-F: | GCCTCCATTCGGGCAAATCTCAG | 121 | Amplification of *NtGCN2* |
| GCN2-R: | CGTCACCAATCGAATTGTGATC |  |  |
| L25-F: | CCCCAAGTACCCTCGTAT | 171 | Amplification of *NtL25* |
| L25-R: | GCTTTCTTCGTCCCATCA |  |  |
